# Effects Induced by Osteophytes on the Strain Distribution in the Vertebral Body Under Different Loading Configurations

**DOI:** 10.3389/fbioe.2021.756609

**Published:** 2021-10-29

**Authors:** Daniele Marras, Marco Palanca, Luca Cristofolini

**Affiliations:** ^1^ Department of Industrial Engineering, Alma Mater Studiorum—Università di Bologna, Bologna, Italy; ^2^ Department of Oncology and Metabolism, INSIGNEO Institute for in silico Medicine, University of Sheffield, Sheffield, United Kingdom

**Keywords:** osteophytes, vertebra, digital image correlation, spine biomechanics, *in vitro* testing

## Abstract

The mechanical consequences of osteophytes are not completely clear. We aimed to understand whether and how the presence of an osteophyte perturbs strain distribution in the neighboring bone. The scope of this study was to evaluate the mechanical behavior induced by the osteophytes using full-field surface strain analysis in different loading configurations. Eight thoracolumbar segments, containing a vertebra with an osteophyte and an adjacent vertebra without an osteophyte (control), were harvested from six human spines. The position and size of the osteophytes were evaluated using clinical computed tomography imaging. The spine segments were biomechanically tested in the elastic regime in different loading configurations while the strains over the frontal and lateral surface of vertebral bodies were measured using digital image correlation. The strain fields in the vertebrae with and without osteophytes were compared. The correlation between osteophyte size and strain alteration was explored. The strain fields measured in the vertebrae with osteophytes were different from the control ones. In pure compression, we observed a mild trend between the size of the osteophyte and the strain distribution (*R* ^2^ = 0.32, *p* = 0.15). A slightly stronger trend was found for bending (*R* ^2^ = 0.44, *p* = 0.075). This study suggests that the osteophytes visibly perturb the strain field in the nearby vertebral area. However, the effect on the surrounding bone is not consistent. Indeed, in some cases the osteophyte shielded the neighboring bone, and in other cases, the osteophyte increased the strains.

## Introduction

Vertebral osteophytes are abnormal bony formations that grow along intervertebral joints (Klaassen et al., 2011). Vertebral osteophytes affect 20–30% of the elderly population (Brown and Neumann 2004). A substantial osteophyte can be found in 20–25% of vertebral columns in people in the age range of 20–45 and in 73–90% of vertebral columns in people aged over 60 years (Nathan 1962). Osteophytes usually originate from the periosteum and typically grow by 4% per annum (Hassett et al., 2003; van der Kraan and van den Berg 2007). Furthermore, osteophytes directly influence the physiologic functions of adjacent organs such as the pharynx, esophagus, lungs, and abdominal aorta (Klaassen et al., 2011). There are several complications associated with osteophyte formation: dysphagia, splanchnic nerve and thoracic aorta compression, and obstructive pneumonia and vena cava obstruction (Nathan, 1987; León et al., 2000; Otake et al., 2002; Chtata et al., 2005; Kanbay et al., 2006).

There is a general consensus on the overall effects of osteophytes on the intervertebral kinematics (Toh et al., 2001; Al-Rawahi et al., 2011; Wagnac et al., 2017). It was observed that vertebral osteophytes seem to stabilize the vertebral column both in bending and pure compression scenarios (Al-Rawahi et al., 2011). They hypothesized that the cross-sectional area of the vertebral body is increased by the presence of the osteophyte- reducing strains (Toh et al., 2001; Al-Rawahi et al., 2011). Furthermore, it was observed that the presence of osteophytes tended to decrease motility in lateral bending and extension, inducing stiffening in spinal segments under different loading configurations (Fujiwara et al., 2000; Tanaka et al., 2001).

Conversely, the mechanical causes of osteophyte formation and growth are not completely clear, and the same applies to the mechanical consequences as well. Some authors suggested that osteophyte formation follows the principles of bony adaptive remodeling (Al-Rawahi et al., 2011; Wagnac et al., 2017). A similar conclusion was supported by combining a quantitative bone remodeling theory with a finite element model (FEM). It was supposed that osteophyte formation is an adaptive process in response to the change of the mechanical environment (He and Xinghua 2006). Furthermore, several studies suggested that the formation of osteophytes is associated with IVD degeneration (Macnab 1971; Vernon-Roberts and Pirie 1977; Heggeness and Doherty 1998; Prescher 1998; Wilke et al., 2006; Pye et al., 2007; Kasai et al., 2009). In addition to the reduction of mobility and nerve compression, IVD degeneration changes the compressive strain distribution and stress concentration (Kumaresan et al., 2001; O’Connell et al., 2011). These studies focused their attention on the strain in the posterior and anterior parts of the annulus fibrosus but did not elucidate what happens to the strain distribution in the bone near the osteophytes.

Further studies used bone remodeling theory to explain kyphosis in the human spine with osteophytes. The strain on the trabecular bone in six human functional spine units (FSUs) was evaluated using texture correlation (Toh et al., 2001). They observed that both the minimum and maximum principal strains were greater during flexion than under axial loading after the removal of the osteophyte. Moreover, the effects of osteophytes on the strain field in the anterior longitudinal ligament (ALL) were measured in different loading scenarios (Palanca et al., 2020). They observed that osteophytes perturb the strain field both in the ALL and in the intervertebral disc (IVD). These studies provide information on the mechanical behavior of the spine, but they did not explain the mechanical local effects induced by the osteophytes in the vertebrae. The investigation of the strain field would enable the understanding of the stress environment and if this can better explain the osteophyte growth later.

The aim of the study was to provide an evaluation of the mechanical behavior induced by osteophytes in the neighboring vertebral bone using full-field surface strain analysis under different loading configurations. First, we hypothesized that the presence of the osteophyte induces a perturbation on the strain field in vertebral bodies. Furthermore, we wanted to test if osteophytes perturb the strain field in a consistent way or not, and if the perturbation is associated with osteophyte features.

## Materials and Methods

### Specimens

Six cadaveric spines were obtained through an ethically approved donation program (Anatomic Gifts Registry, United States), and the tests were performed in accordance with the Declaration of Helsinki. Eight thoracolumbar segments without bony bridges were harvested between the vertebrae (Table 1):

• Seven out of eight specimens consisted of four vertebrae, one with an osteophyte and another without the osteophyte (referred to as “control”) in the middle. In some specimens, the vertebra with the osteophyte was cranial (specimens #4, #7, and #8) while in other specimens it was caudal (specimens # 1, #2, #5, and #6), with respect to the control vertebra.
• One specimen (#3) consisted of five vertebrae, for reasons related to a different study. In this case, the vertebra with the osteophyte was located between two vertebrae that could be used as the control: In this specimen, the caudal adjacent vertebra was chosen as the control.

**TABLE 1:** Details of the eight spinal specimens extracted from six vertebral columns. For each specimen were reported both osteophyte size and the osteophyte–to-control ratio associated with different loading configurations. Two of the donors (B and E) provided two samples each (2 and 3 from B, and 6 and 7 from E).

										Osteophyte-to-control ratio		
ID	Donor	Age	Sex	Height (cm)	Weight (kg)	Segment	Osteophyte position	Osteophyte shape	Osteophyte size (mm)	Pure compression	Anterior bending	Lateral bending
1	A	81	M	182	77	T12—L3	L2-lateral-right	Claw spur	6.24	0.53	—	0.24
2	B	82	F	157	44	T5—T8	T7-frontal-left	Claw spur	4.12	1.92	1.15	—
3	—	—	—	—	—	L1—L5	L3-frontal-left	Traction spur	2.34	0.43	0.37	—
4	C	55	F	165	47	T12—L3	L1-frontal-right	Claw spur	4.03	1.44	1.86	—
5	D	78	M	182	54	T12—L3	L2-frontal-left	Claw spur	15.60	2.93	2.25	—
6	E	51	F	178	130	T11—L2	L1-frontal-right	Claw spur	9.42	3.07	1.39	—
7	—	—	—	—	—	L3—S	L4-frontal-right	Claw spur	11.57	1.03	2.13	—
8	F	73	F	175	72	T10—L1	T11-lateral-left	Claw spur	6.82	0.38	—	0.50

The ALL and the periosteum were removed to expose the cortical bone. Consequently, the segments were aligned (Danesi et al., 2014), and the most caudal and cranial vertebrae were embedded in polymethylmethacrylate (PMMA) bases, to be mounted onto the testing machine.

### Localization of the Osteophytes

All segments were scanned in a quantitative computed tomography (qCT) scanner (Aquilion ONE, Toshiba, Japan) following a bone protocol (current: 200 mA, voltage: 120 KVp, slice thickness: 1 mm, and in-plane resolution: around 0.45 mm). Signs of bony defects, previous fractures, or metastases were excluded.

**FIGURE 1:**
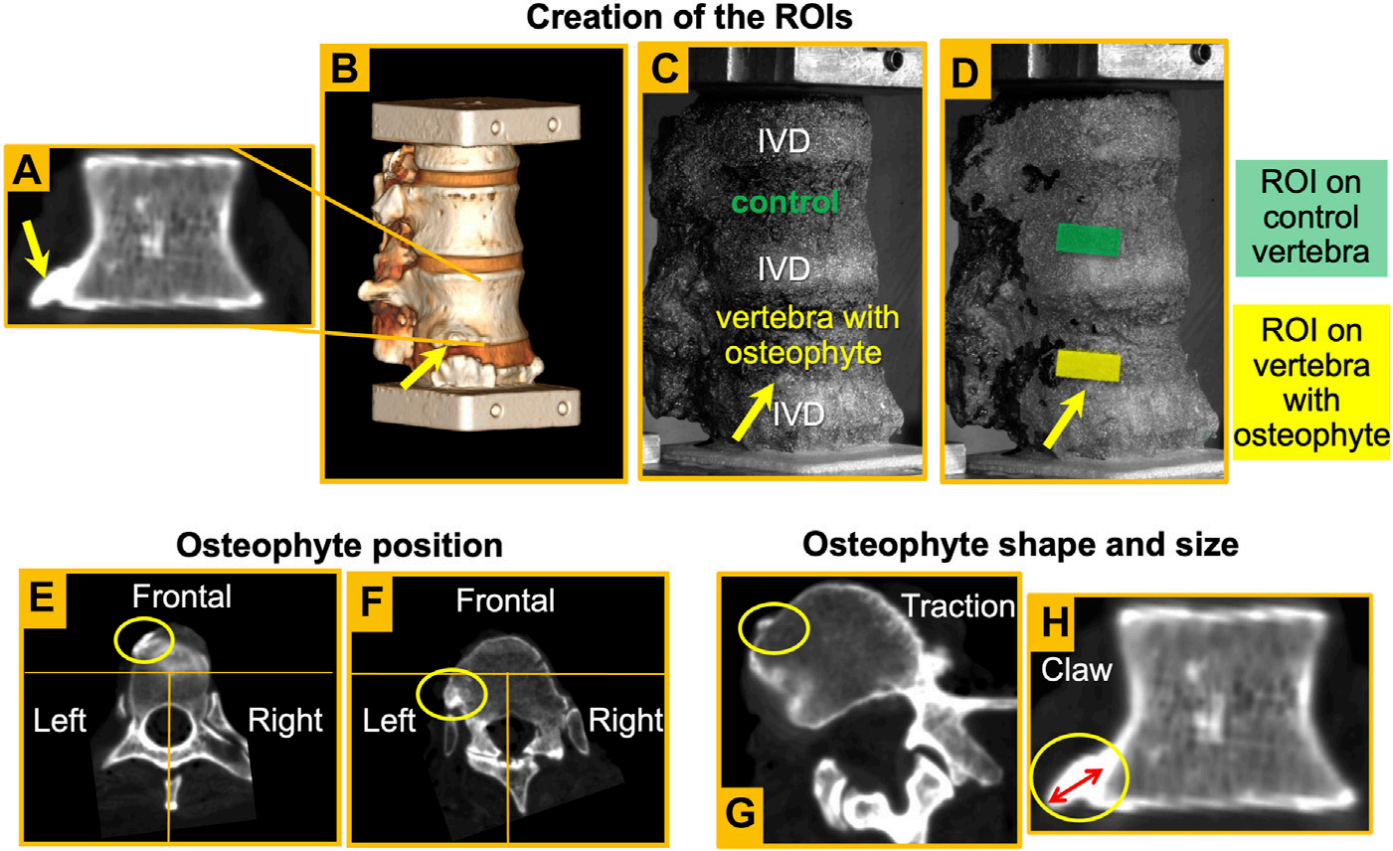
*–* Coronal slice of an L1 vertebra with an osteophyte **(A).** Shape and size of the osteophyte were assessed from these views. Reconstruction of the 3D rendering **(B)** and detection of the osteophyte in the image taken by the DIC system (yellow arrow) **(C).** Creation of the ROIs both in the vertebra with the osteophyte and in the control vertebra **(D)**. Definition of the osteophyte position: examples of an osteophyte in the frontal area **(E)** and one on the left **(F)**. Definition of osteophyte shape: examples of a traction spur **(G)** and a claw osteophyte **(H)**. Definition of osteophyte size (H) according to the method described by Wilke et al. (2006).

The following details of the osteophyte were detected from the qCT scans:

• The position was identified splitting in three areas the CT cross-section of the vertebral body passing through the osteophyte. If the osteophyte was in the anterior area, the position was defined “frontal” or else “lateral” (Figure 1).
• The shape: osteophytes were classified into “claw spur” and “traction spur” (Figure 1), according to Macnab’s classification (Macnab 1971),
• The size of the osteophyte was measured along the long axis of the osteophyte, from the original border of the vertebral body to the tip of the osteophyte, according to the method described by Wilke et al. (2006) (Table 1; Figure 1).

### Mechanical Testing Apparatus and Digital Image Correlation

**FIGURE 2:**
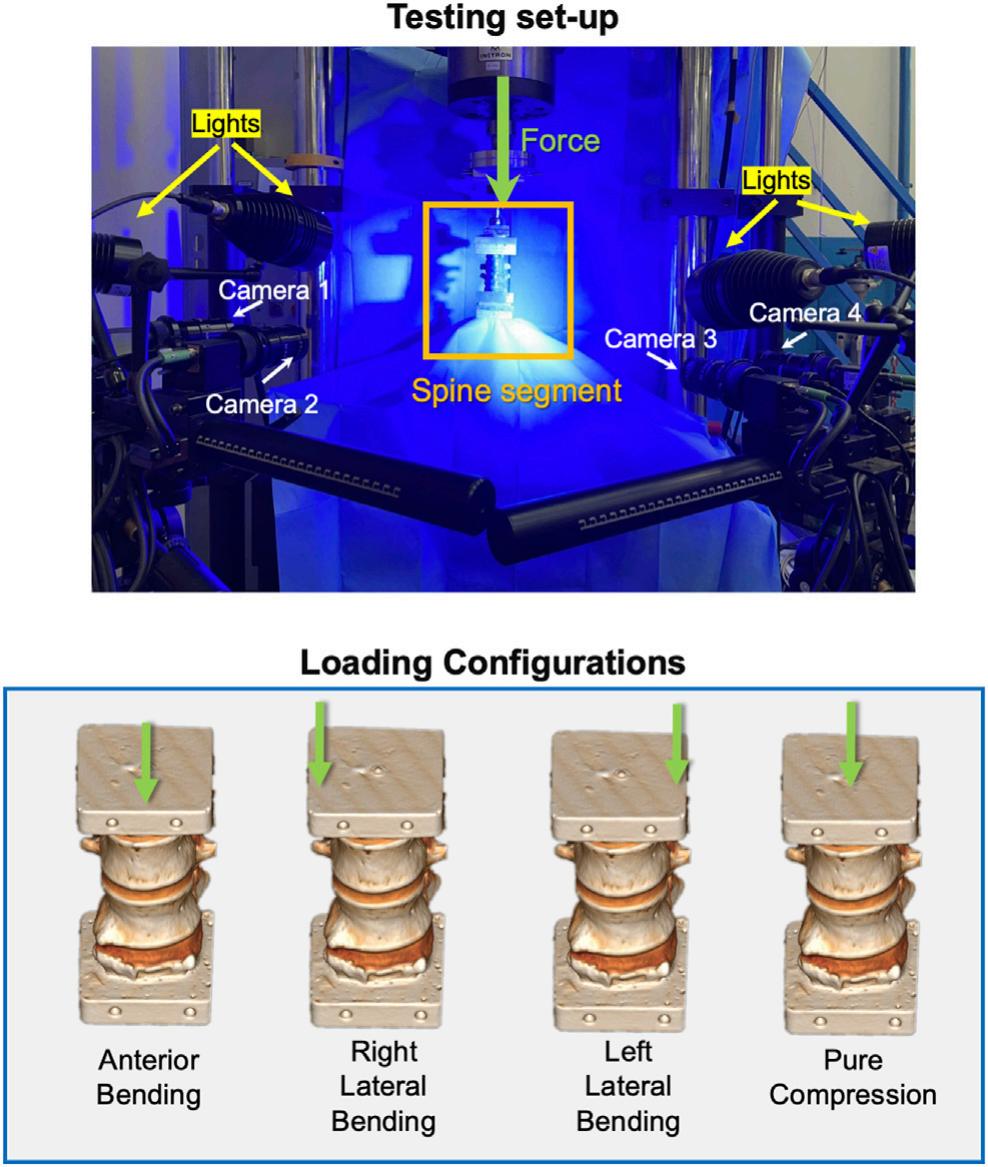
Top: the loading setup and the DIC system. Bottom: the loading configurations applied to the specimens. The green arrows show the vertical force delivered by the actuator: an offset toward anterior, right, or left was used to generate anterior, right, or left bending.

A uniaxial testing machine (Instron 8,500 with a 25 kN load cell, Instron, United Kingdom) was used. The lower vertebra was fixed to the load cell, while the upper vertebra was loaded by the actuator (Figure 2). Each segment was tested under two loading configurations:

• Pure compression was obtained imposing a pure translation of the cranial vertebra (which was rigidly connected to the actuator) toward the caudal one. All other components of translation and rotation were constrained.
• Anterior and lateral bending were obtained applying a vertical force to the cranial vertebra with an anterior or lateral offset. As the force was delivered through low-friction orthogonal linear bearings and a ball joint, the upper vertebra was free to translate in a transverse plane and rotate around all axes. Anterior bending was obtained with an anterior offset equal to 10% of the antero-posterior dimension of the middle IVD. For left and right lateral bending, the offset amounted to 10% of the left–right size of the middle IVD (Palanca et al., 2018). In both cases, the dimension of the middle IVD was measured on the CT scan. The direction of bending depended on the osteophyte position: if the osteophyte was on the anterior side, the specimen was loaded via anterior bending or else via lateral (right or left) bending (Figure 2).

In order to test the different specimens (thoracic or lumbar sections, obtained from different donors) under comparable loading conditions, the applied force was tuned for each specimen. Before the actual test, each specimen was loaded (L_physio_) onto the testing machine in displacement control until an average strain target of 2,500/3,500 microstrain was reached in the anterior part of the control vertebra for compression and anterior bending and in the lateral part for lateral bending. The choice of this range corresponds to the strain levels measured during physiologic motor tasks (Lanyon 1987). In this way, the specimen was loaded onto the elastic regime, thus avoiding bone damage and allowing multiple tests on the same specimen. Ten preconditioning cycles were imposed between 0 and half-L_physio_. Then, the specimens were loaded with monotonic ramp to reach the L_physio_ in 1.0 s. This loading was repeated thrice to assess the tests’ repeatability, and the second one is reported in this article.

In order to measure the full-field minimum principal strain on the external surface of vertebral bodies, the 3D-DIC system (Aramis Adjustable 12M, GOM, Braunschweig, Germany) optimized for spine tests was used (Palanca et al., 2018). The DIC system was set up with four 12 Mpixel cameras (4,096*3,000 pixels) equipped with four 75-mm lenses (Titanar B 75, f4.5). The specimens were illuminated using a LED light system (LED lights with a 10 light cone) (Figure 2). Two measurement volumes were defined to optimize the acquisition for both the smaller specimens (100*80*80 mm^3^) and larger specimens (180*130*130 mm^3^), obtaining a pixel size of 0.03 and 0.04 mm, respectively. Calibration was performed for each new test using two proprietary calibration targets, namely, CP40/MV/100 and CP40/MV/200 (GOM, Braunschweig, Germany). Five images of the unloaded specimen were acquired to evaluate the measurement uncertainties (Palanca et al., 2018). The images were acquired at 25 Hz during the load cycles to measure the full-field strains.

### Definition of the Region of Interest

A rectangular region of interest (ROI) was defined to evaluate how the osteophyte locally affects either the cranial or the caudal area of the vertebral body next to the osteophyte (Figure 1). The height of the ROI was 25% that of the height of the vertebra with the osteophyte, and the width was double the height of the ROI. Such rectangular areas ranged from 48.36 to 124.19 mm^2^ (depending on the size of the respective vertebra with the osteophyte) (standard deviation = 23.97). Among the vertebrae with osteophytes, seven out of eight specimens had the osteophyte in the cranial vertebral endplate. Only specimen #6 had the osteophyte in the caudal vertebral endplate. If the osteophyte was in the superior vertebral endplate, the ROI was caudal compared to the osteophyte and the upper long side of the ROI placed under the osteophyte region. By contrast, if the osteophyte was in the inferior vertebral endplate, the ROI was cranial compared to the osteophyte and the lower long side of the ROI placed immediately above the osteophyte region to center the rectangular ROI on the vertebral body near the osteophyte, following the previous reasoning. Once the ROI on the vertebra with the osteophyte had been defined, a corresponding ROI was created on the control vertebra in the same relative position. The control ROI had the same size as the ROI on the vertebra with the osteophyte (Figure 1). Only bony surfaces were included in the ROIs, excluding adjacent IVDs (Palanca et al., 2018).

The average of the minimum principal strain was computed for the two ROIs. The osteophyte-to-control ratio was used to normalize the strain of each ROI near the osteophyte with respect to the control ROI. The osteophyte-to-control ratio was defined as the ratio between the average minimum principal strain of the ROI on the vertebra with the osteophyte and the average minimum principal strain of the control ROI. If the osteophyte-to-control ratio is higher than 1.00, the ROI on the vertebra with the osteophyte is more deformed than the control ROI. By contrast, if the osteophyte-to-control ratio is lower than 1.00, the control ROI is more deformed than the ROI on the vertebra with the osteophyte.

### Data Processing and Statistics

For each specimen, the size of the osteophyte, the average of minimum principal strain in both ROIs, and the osteophyte-to-control ratio were evaluated.

To confirm that the ROIs on the vertebra with the osteophyte and the ROIs of the control vertebra of each specimen had different behaviors, the respective fields of minimum principal strains were compared using the Kolmogorov–Smirnov test.

To assess the significance of the difference between the average strains in the ROIs near the osteophyte and in the ROIs of the control vertebrae, the averages of such ROIs were compared using *t*-test to ascertain if data were normal and homoscedastic or the Wilcoxon signed-rank test if data were not normally distributed, for each loading scenario. Normal distribution and homoscedasticity were verified, respectively, using the Shapiro–Wilk test and Levene’s test.

The strength of the association between the size of the osteophytes and the osteophyte-to-control ratio was evaluated using linear regression analyses, separately for pure compression and bending.

All statistical analyses were performed using Matlab (R2020a, The Mathworks, Inc., Natwick, MA, United States).

## Results

**FIGURE 3:**
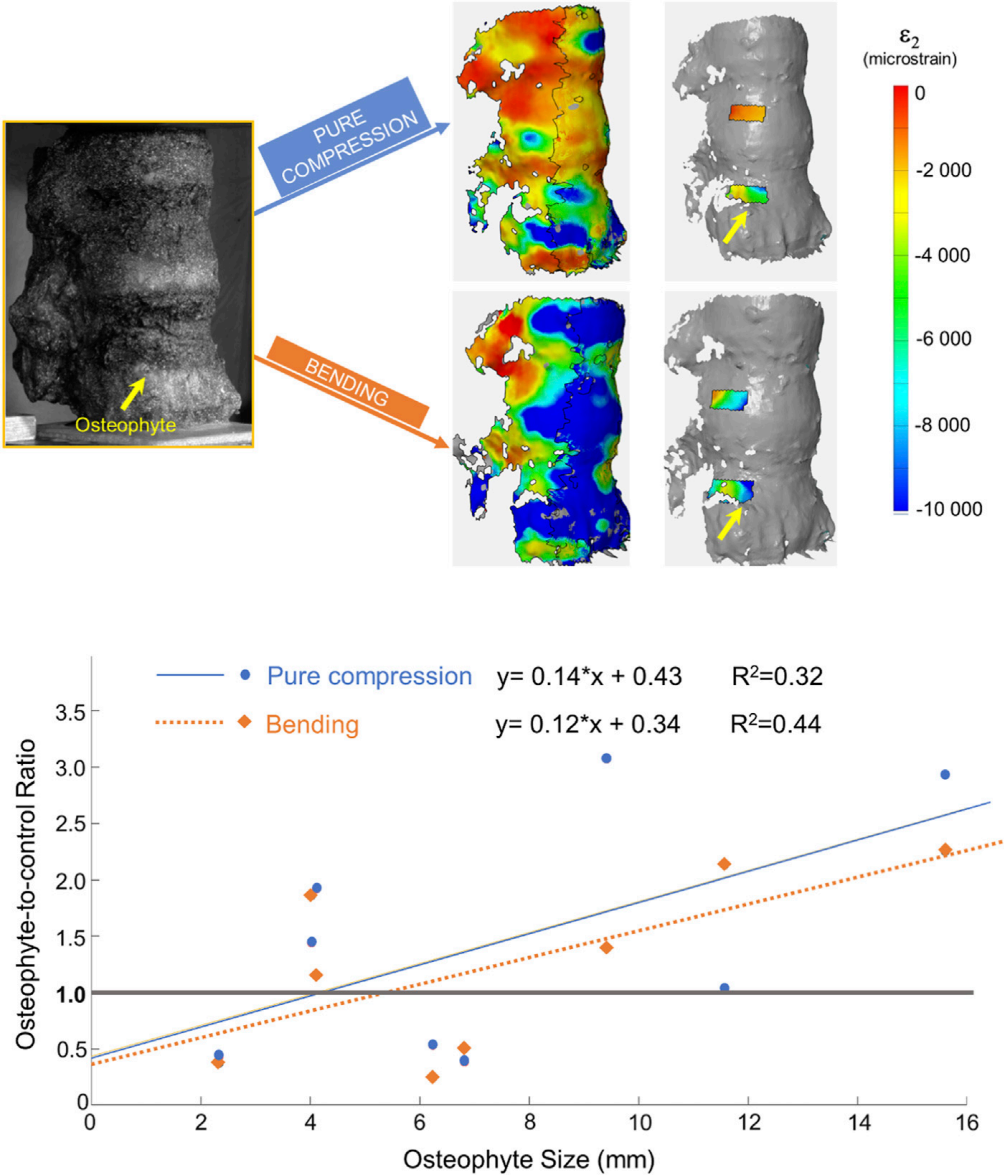
Top: from left to right: image taken by the DIC cameras, full-field minimum principal strains, and minimum principal strain measured in the ROIs for the two loading configurations. In all the images, the specimen is viewed from the right-anterior side. The yellow arrow highlights the position of the osteophyte. Bottom: plot and linear regressions between the osteophyte-to-control ratio and the osteophyte size under two different loading configurations. The gray line in the graph depicts the condition in which the two ROIs in the same specimen have the same deformation.

All tests were successfully performed with no visible damage to the specimens. The DIC-measured strain had a systematic error of 30 microstrain or less and a random error below 100 microstrain. The minimum principal strain maps were measured in all ROIs (control and with osteophyte) for each loading configuration (Figure 3).

The osteophyte perturbed the strain field in the neighboring surface of the vertebral body. Indeed, the strain field in the ROI near the osteophyte and the control ROI of the same specimen, under different loading conditions, was significantly different (Kolmogorov– Smirnov test, *p* < 0.0001).

By contrast, the value of the average strains over the ROIs with the osteophyte and over the control ROIs were not significantly different, and both were under pure compression (Wilcoxon signed-rank test, *p* = 0.46) and under bending (*t*-test, *p* = 0.96).

Seven out of eight specimens had an osteophyte-to-control ratio different from 1.00, both in bending and in pure compression scenarios (Table 1). Only specimen #7 showed an osteophyte-to-control ratio very close to the one (1.03) for anterior bending, whereas it was 2.13 for pure compression. Under pure compression, a mild trend between the osteophyte-to-control ratio and the size of the osteophyte was observed (*R* ^2^ = 0.32; Figure 3), which was not statistically significant (*p*-value = 0.15). A slightly higher trend was found for bending (*R* ^2^ = 0.44; Figure 3), but still it was not statistically significant (*p*-value = 0.075).

## Discussion

The first hypothesis of this study was that the presence of the osteophyte affects the strain field. In addition, we wanted to understand whether the mechanical effects of the osteophytes are consistent, or if the osteophyte could either shield the nearby vertebral areas or concentrate the strains. We also aimed to test if a correlation exists between the size of the osteophyte and its mechanical effects.

In this study, eight thoracolumbar spine segments were tested to evaluate the mechanical behavior induced by the presence of osteophytes. Full-field surface strain analyses were performed by means of DIC on the anterior and lateral surface of vertebral bodies. All specimens were tested in pure compression and bending. In order to test the hypotheses, the minimum principal strain maps were measured in all ROIs.

The strain maps showed that the osteophyte perturbed the strain field in the neighboring surface of the vertebral body. In fact, the strain fields in the ROI near the osteophyte and in the control ROI of the same specimen were significantly different (Kolmogorov–Smirnov test, *p* < 0.0001) for both the loading configurations. However, we could not identify a systematic behavior where the osteophyte either consistently shielded the neighboring cortical bone or consistently concentrated the strains. In five out of eight specimens, the average of the minimum principal strain was larger in the ROI with the osteophyte than in the control ROI, both in pure compression and bending (Table 1). This led us to hypothesize that other factors (e.g., the disc degeneration, bone mineral density, and vertebral shape) may contribute to define the behavior of the vertebra with osteophytes.

Most of the studies in the literature were performed to analyze the mechanical behavior of the osteophyte focused on the spine kinematics (Fujiwara et al., 2000; Tanaka et al., 2001; Al-Rawahi et al., 2011; Wagnac et al., 2017). To the best authors’ knowledge, this is the first study where the strain field on the bone surface near the osteophytes was directly measured. Thus, it is difficult to directly compare our study with other studies.

A previous study investigated the strain in the trabecular bone with osteophytes and after osteophytes removal. They cut a slice from six thoracic FSUs and loaded them in two configurations (anterior bending and axial loading) (Toh et al., 2001). They observed osteophyte-induced perturbations of the strain field in the trabecular bone next to the osteophyte, which is in the same line as our findings. However, they noticed that in anterior bending, the osteophyte tended to shield the trabecular bone more than in axial loading. Conversely, our findings seem to show that there are no differences between these two loading configurations. The differences between our findings and their study may be due to the following facts: 1) a slice of an FSU was tested (Kettler et al., 2000; Dickey and Kerr 2003) and 2) the effects of osteophytes that form bony bridges were explored. Moreover, Toh et al. (2001) performed a physical removal of the osteophyte and showed that as the osteophyte was removed, the trabecular strains were lightly more concentrated toward the anterior cortex than the specimen with the osteophyte. In our study, considering the different type of osteophytes, we chose to leave the bone structure intact. Indeed, the bone follows an adaptation that lasts for years (Turner and Akhter 1999). Thus, the region surrounding the osteophytes is adapted to bear a load distribution influenced by the presence of the osteophyte. The removal of the osteophyte could result in a condition never experienced by the vertebra and thus is not relevant. In addition, the cortical shell and the osteophyte are merged as a single body, without a clear distinction (at least at the level of the qCT scans). Thus, shaving of the osteophyte would be quite arbitrary. By contrast, an analysis of the strain distribution in the trabecular bone behind the osteophyte could be performed using a digital volume correlation approach (Roberts et al., 2014).

Another study explored the mechanical behavior of the ALL through DIC strain measurement (Palanca et al., 2020). They indirectly observed an alteration of the strain field in correspondence of osteophytes for all loading configurations except left lateral bending. They showed that osteophytes perturb the strain field both in the ALL and in the IVD. Their observations are consistent with our findings, confirming that osteophytes induce an intensification of the strain in their nearby regions.

In our study, a fracture was not reached as the same specimens were preserved for additional tests later (not presented here). The small sample size is an important limitation of this study. As we wanted to minimize the uncertainty caused by the inter-subject variability, we tested spine segments with a vertebra with osteophyte and an adjacent control vertebra. This enabled direct paired comparisons, thus increasing statistical power. The selection criteria focused on comparable osteophytes, for example, excluding segments with bony bridges. However, both the spine segments (from T5-T8 to L3-S1) and the shape and location of the osteophytes varied. In order to test the different specimens under similar strain levels, a validated protocol was implemented (Danesi et al., 2014; Palanca et al., 2021) to load the different segments in a comparable way. Loading configurations mimicking basic physiologic loads were performed. Our tests were not focused on analyzing complex motor tasks because we wanted to evaluate single loading components in order to have higher control on the experiment. The precision and accuracy of the DIC system was optimized for each acquisition; however, testing fresh specimens involved leakages of biological fluid that can cause lack of local correlation (Palanca et al., 2018). Nevertheless, the entire acquisition allowed to distinctly express what happened in different specimens for different loading configurations.

The preliminary results of this study seem to suggest that the osteophyte significantly perturbed the strain distribution in the neighboring surface of the vertebral body. Nevertheless, how osteophytes perturb the surrounding area is not consistent. In some specimens the osteophyte concentrated the strains in the neighboring bone, while in others the strains were reduced. Our findings could be the starting point for further studies to evaluate if the osteophytes are either a degenerative or an adaptive condition.

## Data Availability

The original contributions presented in the study are included in the article/Supplementary Material; further inquiries can be directed to the corresponding author.
