# Supplementary material for: Effects Induced by Osteophytes on the Strain Distribution in the Vertebral Body Under Different Loading Configurations
Source: Front Bioeng Biotechnol. 2021 Oct 29;9:756609. doi: 10.3389/fbioe.2021.756609 (PMC8585771; doi:10.3389/fbioe.2021.756609)

Donor A
Specimen #1
T12 - L3

Control vertebra:
L1

Position of the osteophyte:
L2 – Lateral - Right

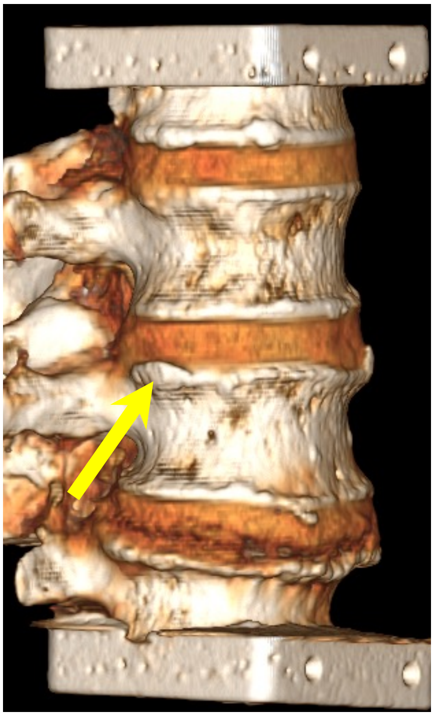

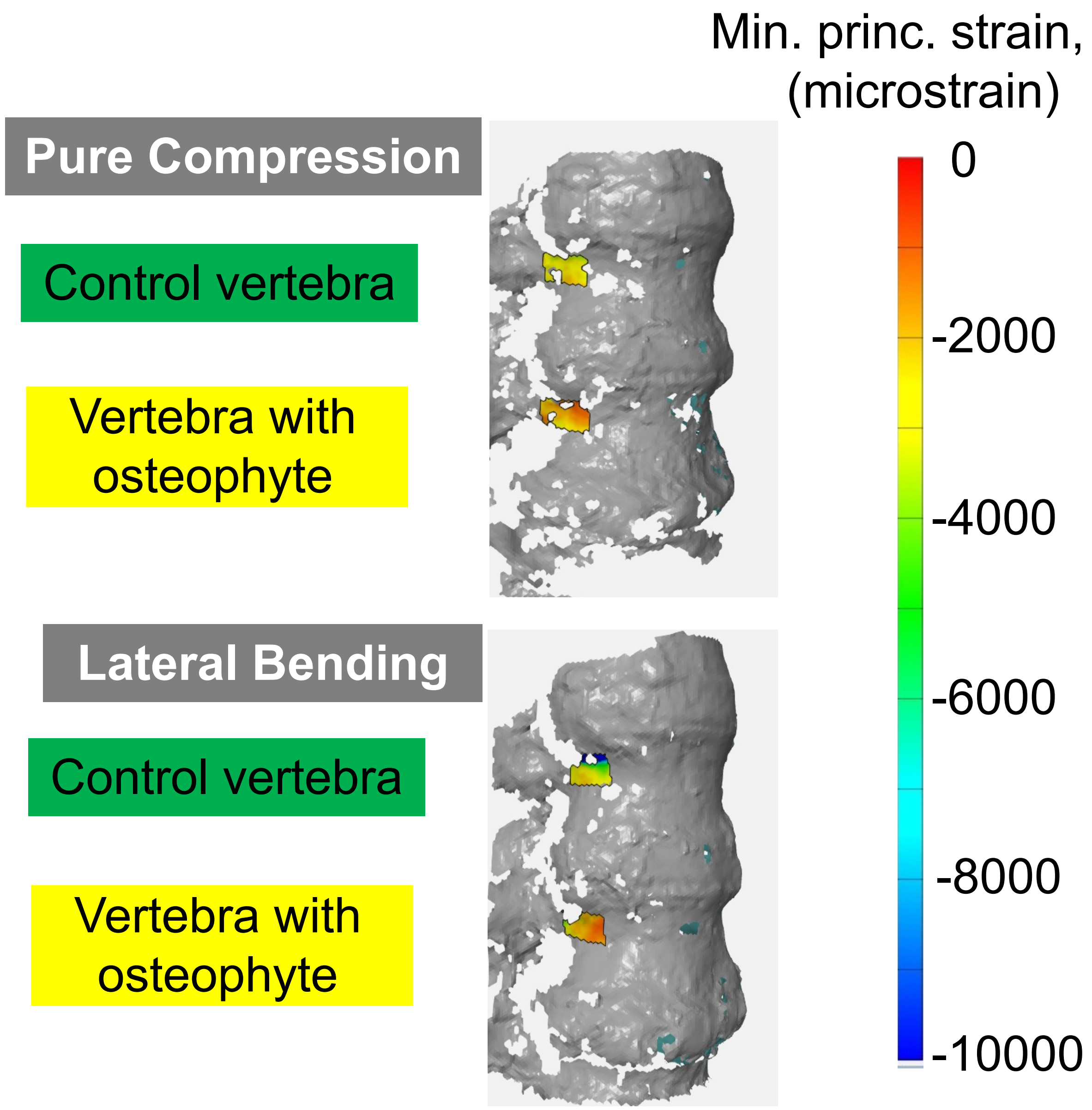

Donor B
Specimen #2
T5 - T8

Control vertebra:
T6

Position of the osteophyte:
T7 – Frontal - Left

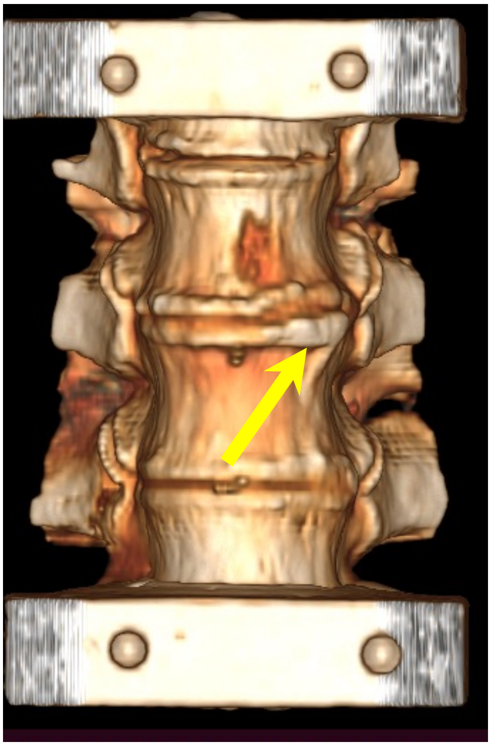

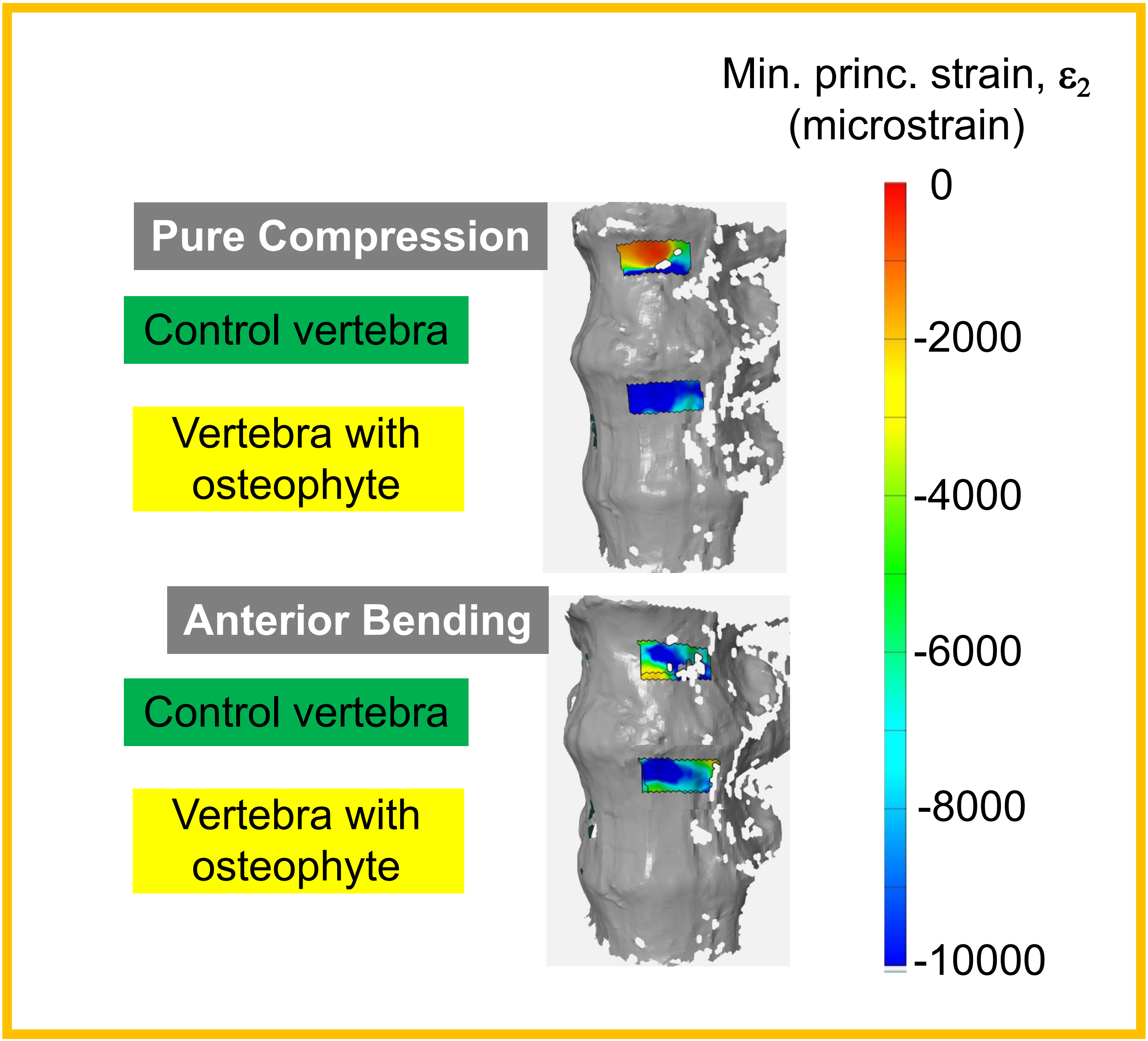

Donor B
Specimen #3
L1 - L5

Control vertebra:
L4

Position of the osteophyte:
L3 – Frontal - Left

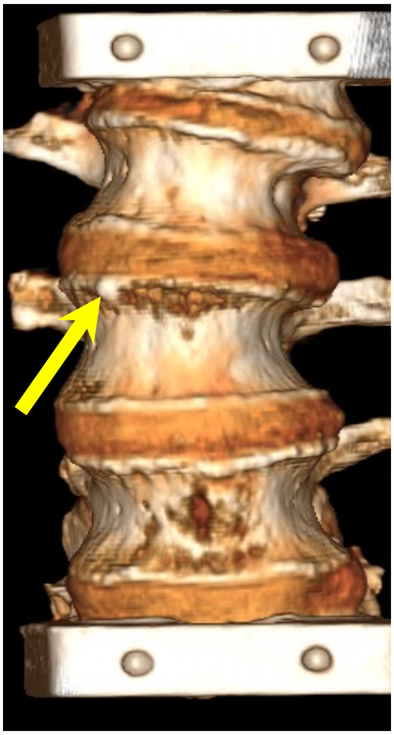

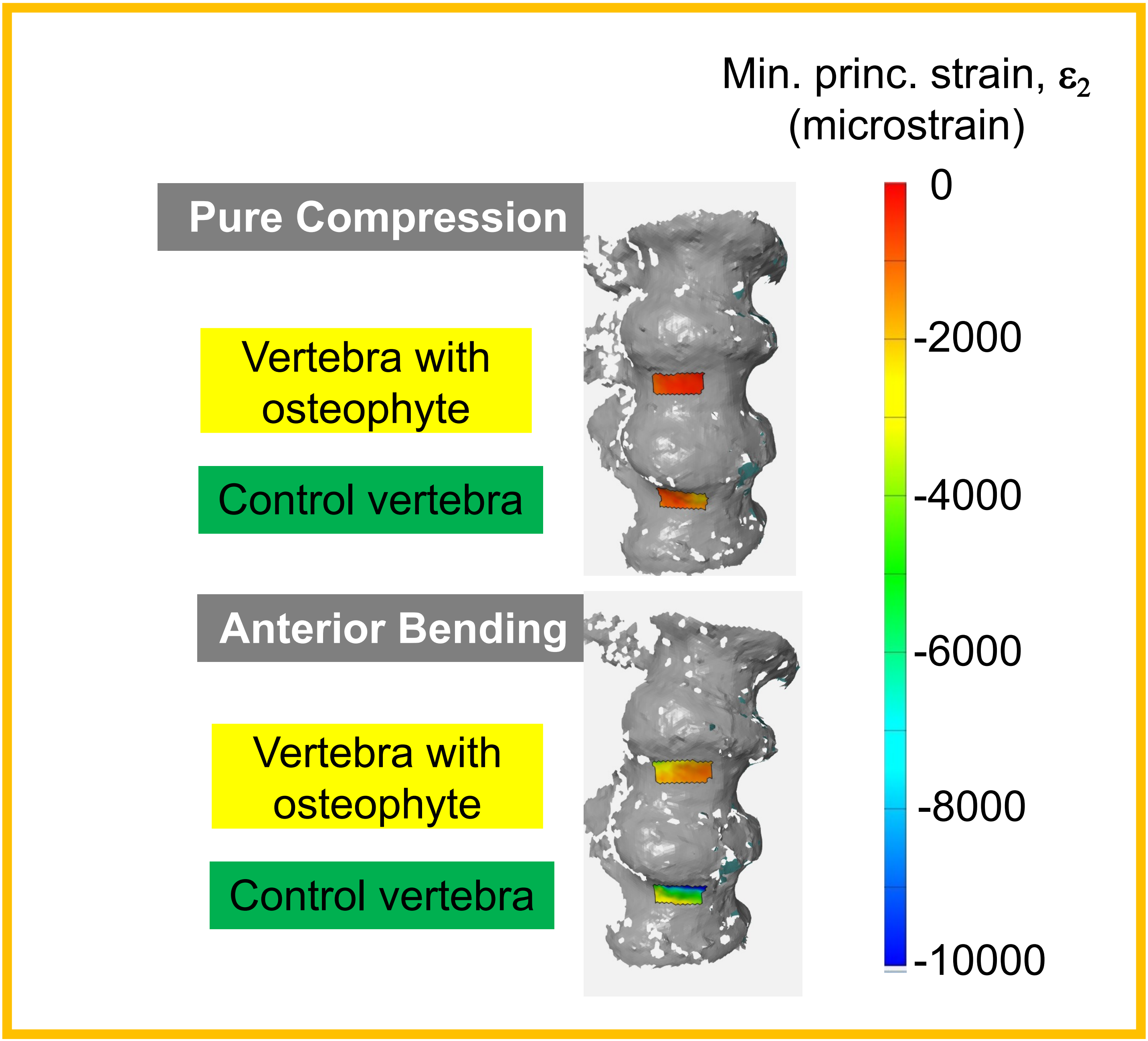

Donor C
Specimen #4
T12 - L3

Control vertebra:
L2

Position of the osteophyte:
L1 – Frontal - Right

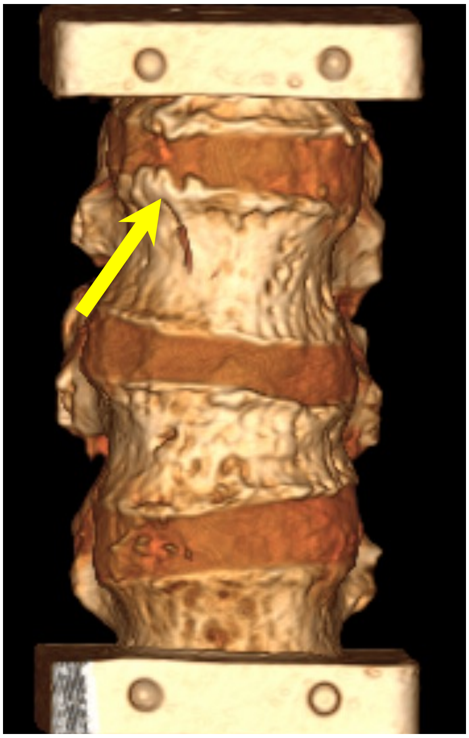

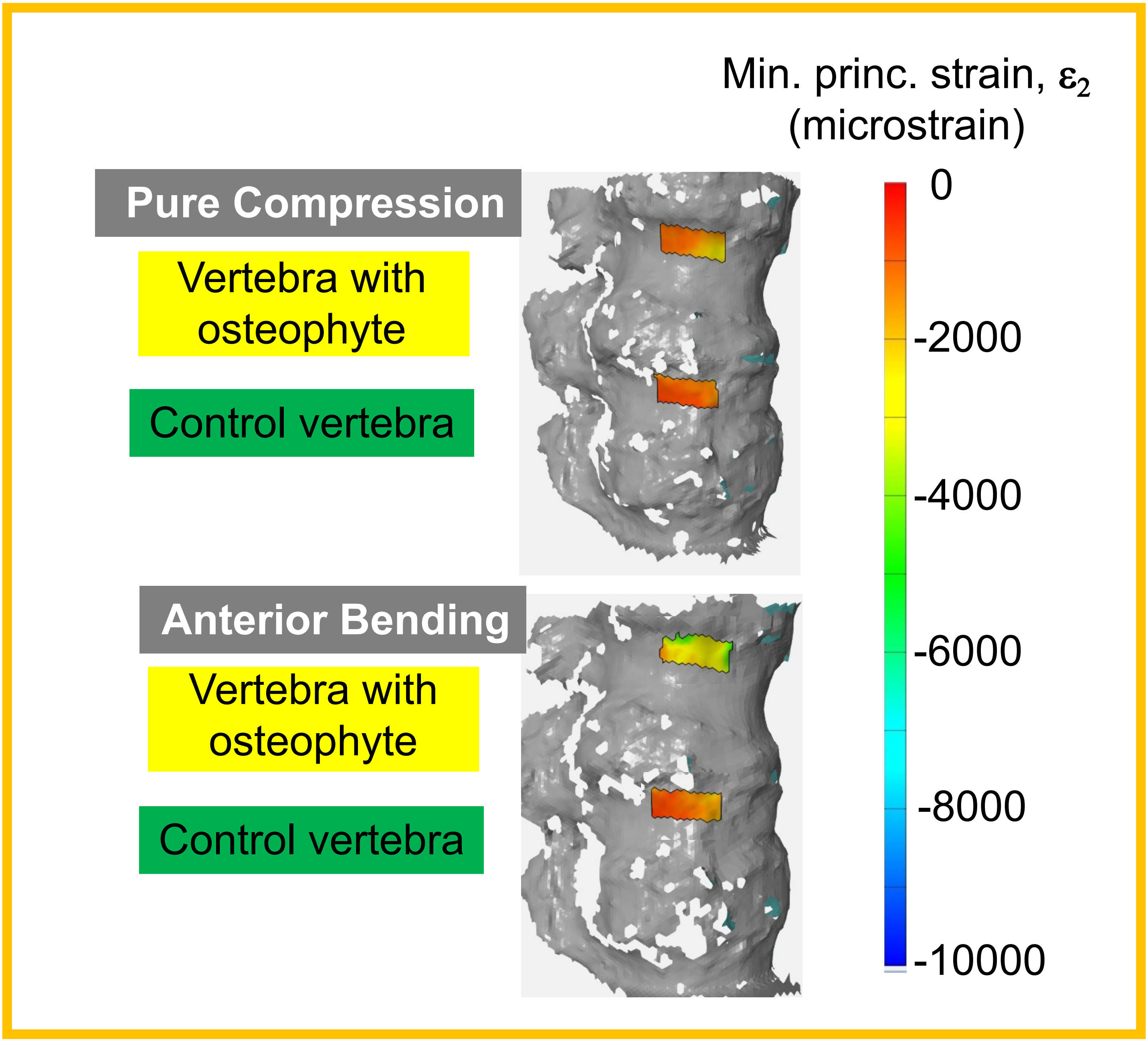

Donor D
Specimen #5
T12 - L3

Control vertebra:
L1

Position of the osteophyte:
L2 – Frontal - Right

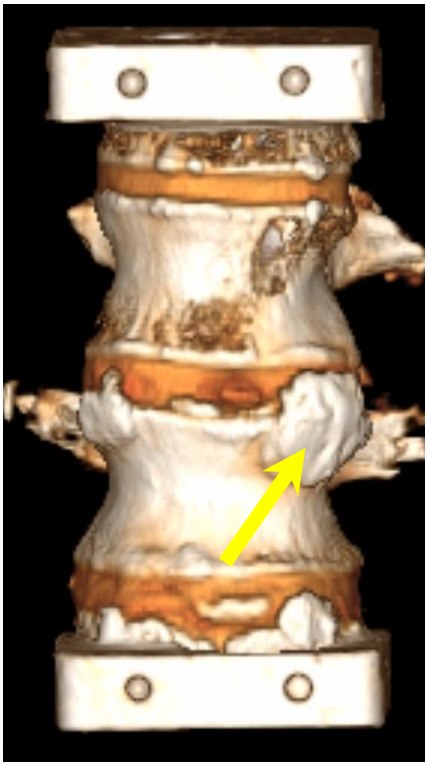

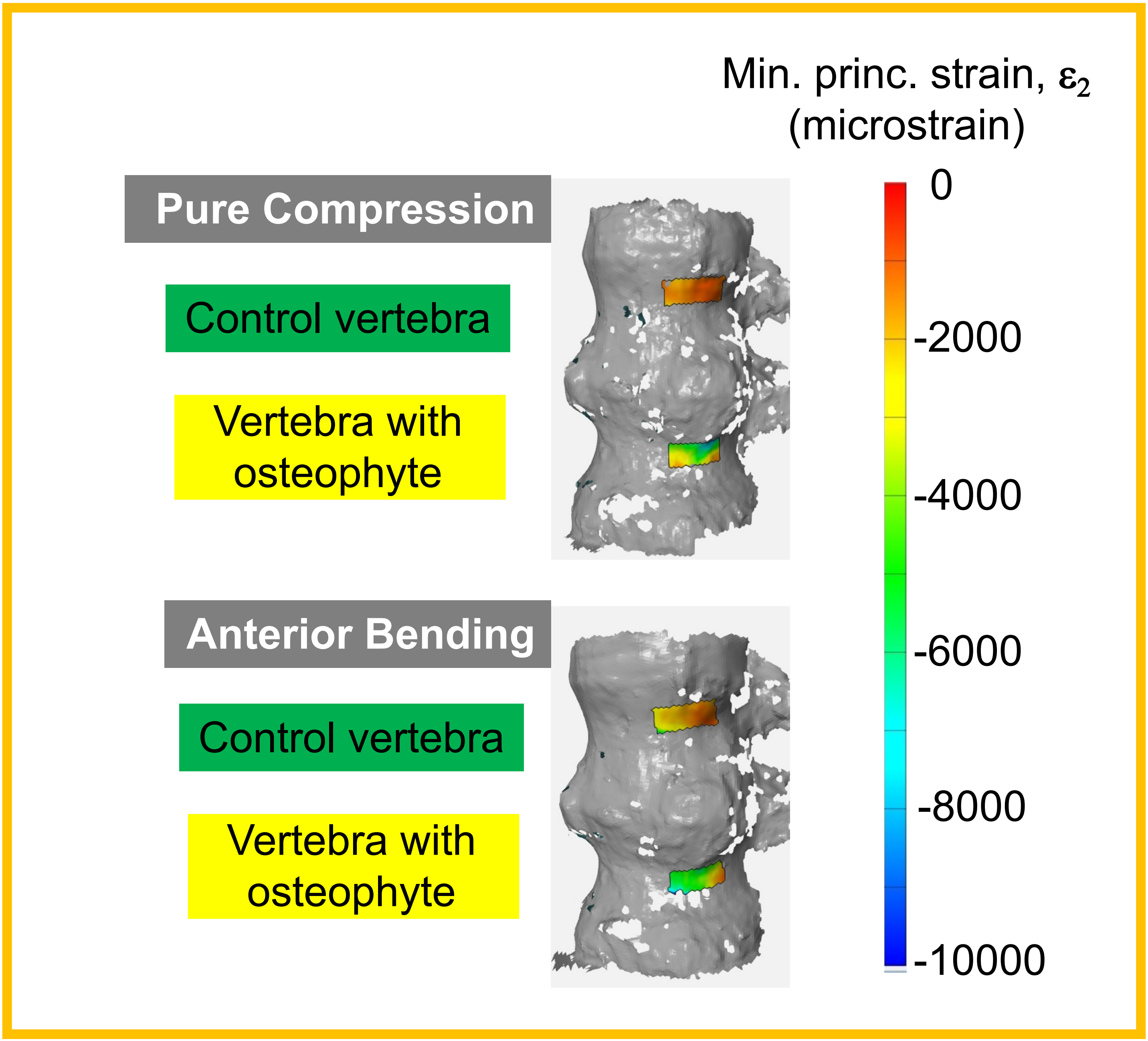

Donor E
Specimen #6
T11 - L2

Control vertebra:
T12

Position of the osteophyte:
L1 – Frontal - Right

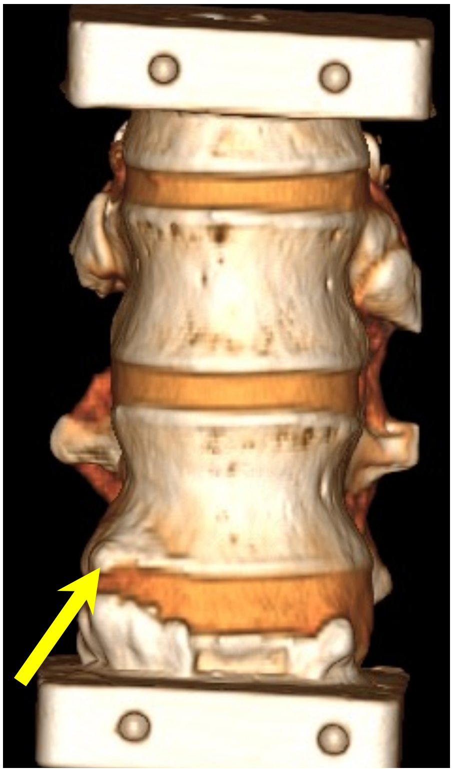

Pure Compression

Control vertebra

Vertebra with osteophyte

Anterior Bending

Control vertebra

Vertebra with osteophyte

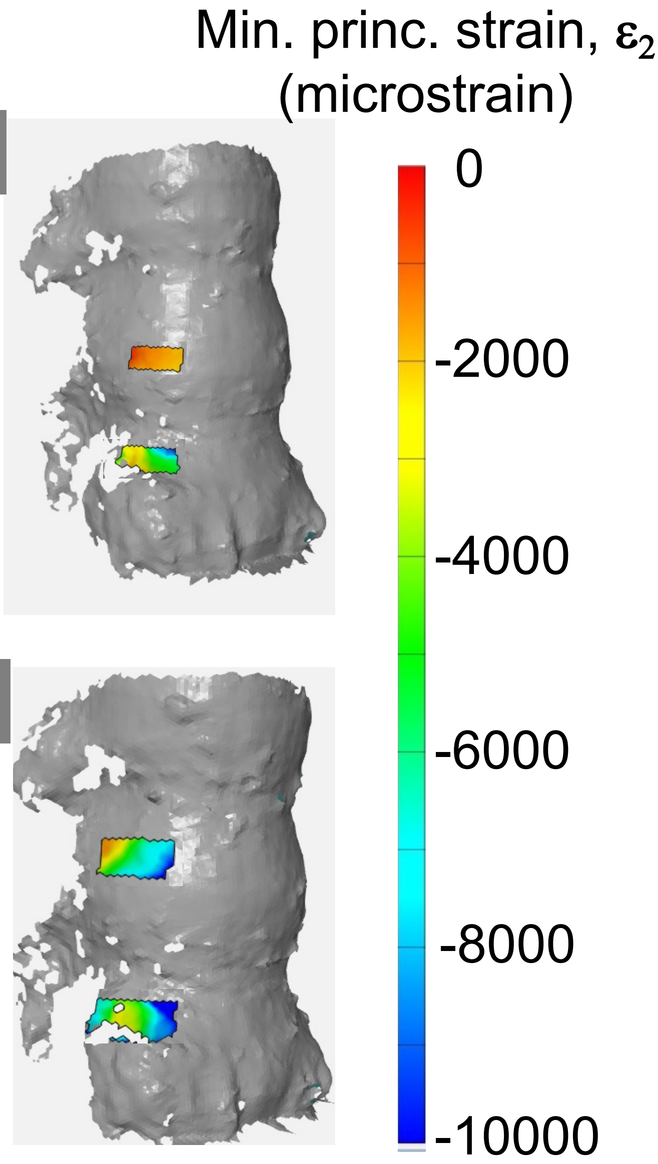

Donor E
Specimen #7
L3 - S1

Control vertebra:
L5

Position of the osteophyte:
L4 – Frontal - Right

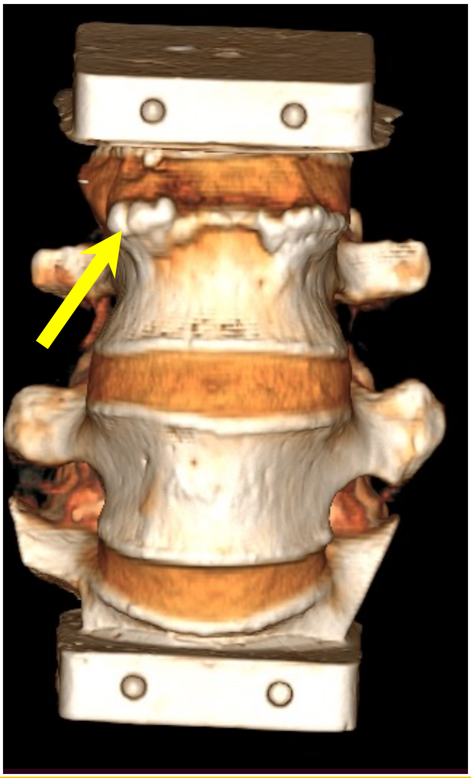

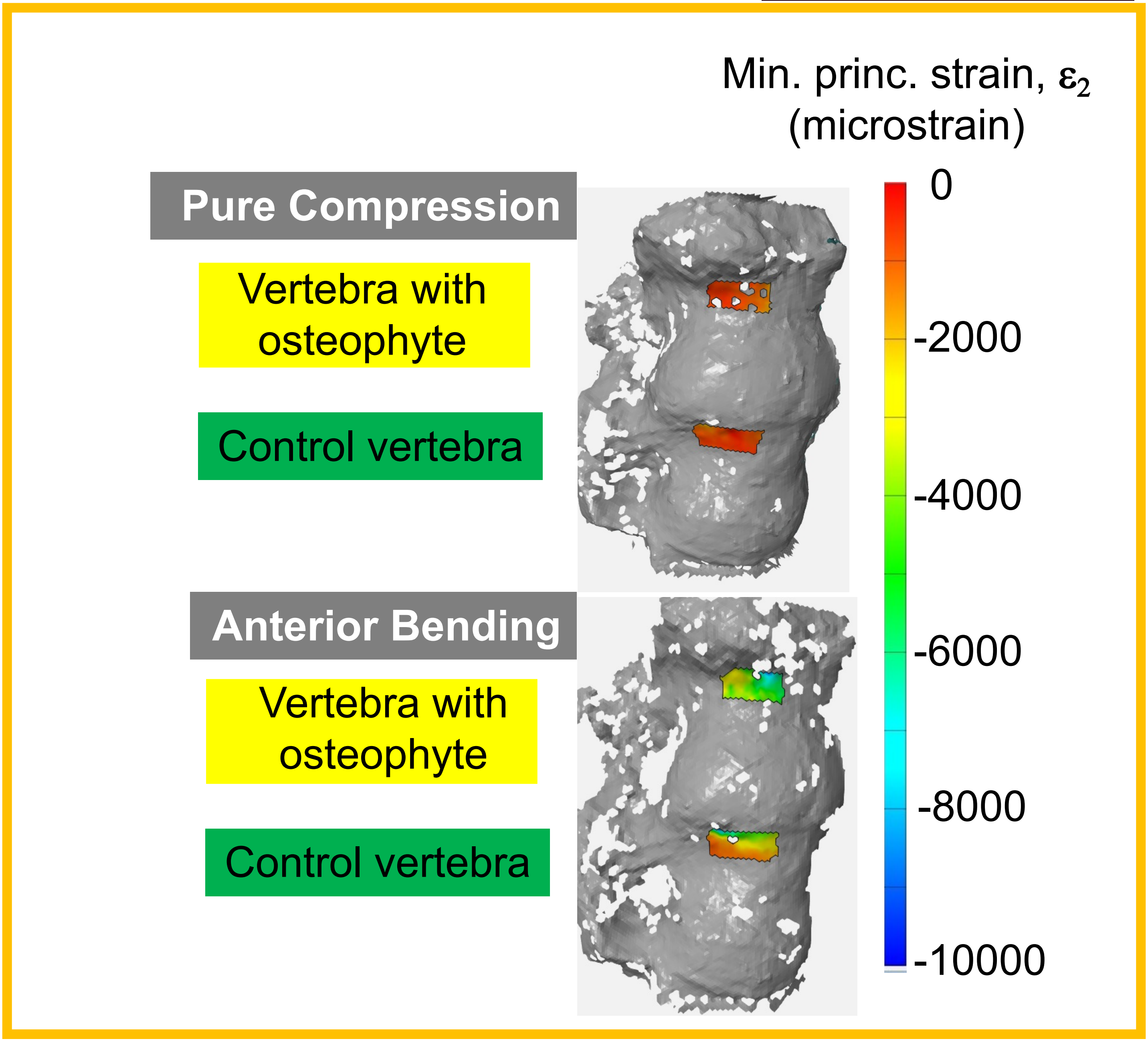

Donor F
Specimen #8
T10 - L1

Control vertebra:
T12

Position of the osteophyte:
T11 – Lateral - Left

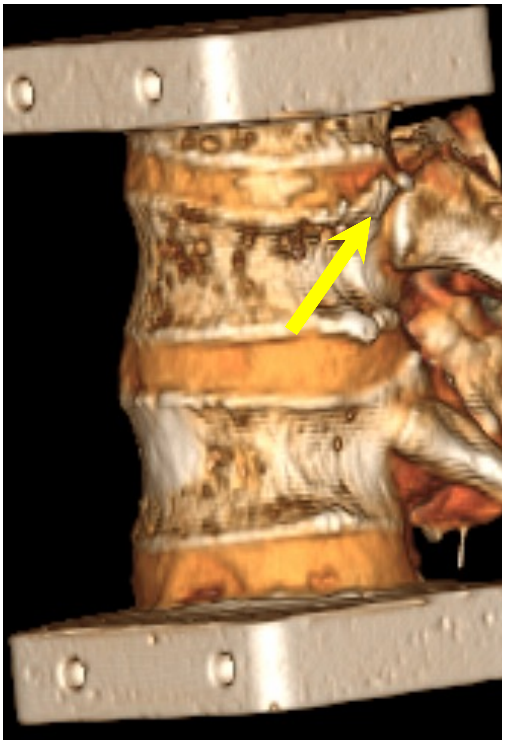

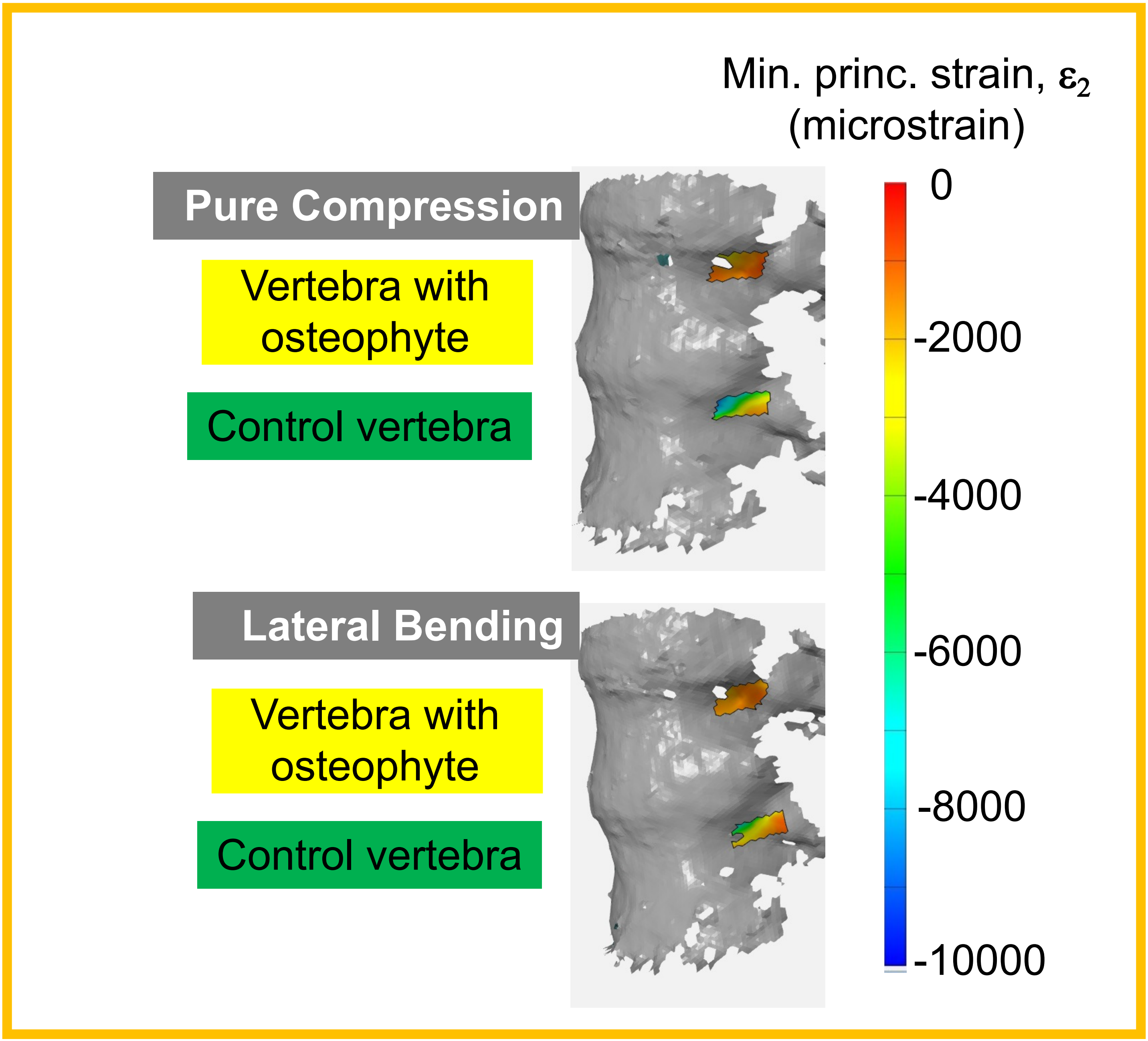

Supplement: Supplementary file 1 [file DataSheet1.PDF]
